# The Differential Outcomes Procedure Enhances Adherence to Treatment: A Simulated Study with Healthy Adults

**DOI:** 10.3389/fpsyg.2015.01780

**Published:** 2015-11-20

**Authors:** Michael Molina, Victoria Plaza, Luis J. Fuentes, Angeles F. Estévez

**Affiliations:** ^1^Departamento de Psicología, Universidad de Almería, Almería, Spain; ^2^Facultad de Ciencias Sociales y Humanidades, Universidad Autónoma de Chile, Talca, Chile; ^3^Departamento de Psicología Básica y Metodología, Regional Campus of Excellence Mare Nostrum, Universidad de Murcia, Murcia, Spain

**Keywords:** differential outcomes procedure, discriminative learning, long-term memory, healthy adults, adherence to treatment

## Abstract

Memory for medical recommendations is a prerequisite for good adherence to treatment, and therefore to ameliorate the negative effects of the disease, a problem that mainly affects people with memory deficits. We conducted a simulated study to test the utility of a procedure (the differential outcomes procedure, DOP) that may improve adherence to treatment by increasing the patient’s learning and retention of medical recommendations regarding medication. The DOP requires the structure of a conditional discriminative learning task in which correct choice responses to specific stimulus–stimulus associations are reinforced with a particular reinforcer or outcome. In two experiments, participants had to learn and retain in their memory the pills that were associated with particular disorders. To assess whether the DOP improved long-term retention of the learned disorder/pill associations, participants were asked to perform two recognition memory tests, 1 h and 1 week after completing the learning phase. The results showed that compared with the standard non-differential outcomes procedure, the DOP produced better learning and long-term retention of the previously learned associations. These findings suggest that the DOP can be used as a useful complementary technique in intervention programs targeted at increasing adherence to clinical recommendations.

## Introduction

The adherence to treatment, that is, the degree to which patients follow medical recommendations, is fundamental to improving health outcomes and quality of life. Medication or drug compliance is a key critical issue, particularly in patient populations, such as the elderly, that have several medications that need to be managed. Lack of adherence to treatment may have dramatic consequences not only for health but also for economy. Patients that do not adhere to the treatment usually show poor control of their diseases, and consequently may require additional drug therapy or even hospitalization (Hughes, 2004). The costs of care due to reduced adherence to treatment may vary among the different countries, but they are high enough as for governments to promote investigation to ameliorate their costly consequences. However, adherence is a multidimensional phenomenon and in certain populations (e.g., the elderly) a compound of factors may underlie non-adherence to the medical recommendations. In fact, according to the World Health Organization (2003), five dimensions determine adherence: (1) social/economic factors, (2) provider-patient/health care system factor, (3) condition-related factors, (4) therapy-related factors, and (5) patient-related factors. A comprehensive analysis of each dimension is beyond the scope of the present study, in which we just focus on patient-related factors. Concretely, we would like to highlight how the complexity of treatments may burden patients’ memory for specific information regarding medical recommendations.

Memory for medical information, about all drug treatments, is a prerequisite for good adherence to the treatment, and therefore to ameliorate the negative effects of the disease. However, up to 80% of information provided by the clinician may be forgotten almost immediately and recall is rather inaccurate, especially when the patients are old or anxious (see Kessels, 2003, for a review). In a study conducted with type 2 diabetes patients, nearly half of them mentioned forgetfulness as one of the major non-intentional reasons for non-adherence to the treatment (Adisa et al., 2009).

Loss of episodic information is frequently associated with age-related cognitive deficits (e.g., working memory). Thus, it is not surprising that elderly people may have more difficulties to retain and follow medical recommendations than younger people. In fact, the issue of non-adherence increases notably in older patients who have multiple morbidities (National Institute for Health and Care Excellence, 2015), which in turn requires managing multiple drugs and hence more memory resources. Both impaired memory and a high number of medicines commonly prescribed to older patients may explain the observed low adherence to medication that characterizes this population (Kripalani et al., 2007), above all when patients suffer from three or more morbidities and have to take three or more drugs (Tavares et al., 2013). Given that the population is aging and life expectancy is increasing, any procedure that helps patients to overcome their memory loss concerning medical treatment would foster their adherence to medical prescriptions and subsequent health improvement.

Sometimes applied science may benefit from basic science, and in some of our previous research we have demonstrated the potential of adapting procedures coming from experimental psychology to ameliorate learning and/or memory deficits associated with diverse pathological conditions (for a review, see López-Crespo and Estévez, 2013; see also Overmier, 2007). The present research is an example of this. In the following sections we first describe the main characteristics of a procedure imported from animal studies in the field of learning. Then, we present evidence of how that procedure has been adapted to human studies and its potential benefits as a therapeutic technique. Finally, we illustrate experimentally how that procedure may be useful to promote the patient’s adherence to medical recommendations related to treatment with drugs.

### The Differential Outcomes Procedure

The differential outcomes procedure (DOP) applies to conditional discriminative tasks in which a correct choice response to a specific stimulus–stimulus association is reinforced with a unique reinforcer or outcome (Trapold, 1970; Trapold and Overmier, 1972). In a conditional discrimination task a sample stimulus is associated with one of several comparison stimuli. Each particular sample-comparison stimulus association requires a specific response that must first be learned and then reinforced with a determined outcome. Trapold (1970) published the first demonstration of the DOP in rats. When reinforcement of correct choices was arranged according to the DOP rather than the more standard common outcomes procedure, in which only one type of reinforcer is administered, the rate with which rats learned the associations accelerated, and the final accuracy level was higher.

In the last decades several studies have explored the potential usefulness of the DOP as a therapeutic (and/or pedagogic) tool to increase discriminative learning in children (Maki et al., 1995; Estévez et al., 2001; Estévez and Fuentes, 2003; Martínez et al., 2009, 2013) and adults (Miller et al., 2002; Easton, 2004; Estévez et al., 2007; Mok and Overmier, 2007). Importantly, the DOP not only enhances conditional discriminative learning but also memory (Plaza et al., 2011, 2013; see López-Crespo and Estévez, 2013, for a review).

### The DOP in Pathology

The DOP has also facilitated discriminative learning—sometimes very modestly, sometimes dramatically—in different clinical populations, such as autistic children (Hewett, 1965; Litt and Schreibman, 1981), children and adults with mental handicaps (e.g., Saunders and Sailor, 1979; Malanga and Poling, 1992; Joseph et al., 1997), children and adults with Down syndrome (Estévez et al., 2003), and prematurely born children (Martínez et al., 2012).

The results obtained in the aforementioned studies suggest that this procedure may be useful to facilitate the learning of symbolic stimulus–stimulus relationships. However, there is also ample evidence that demonstrates the DOP benefits on memory-based performance in individuals with memory complaints. For instance, Hochhalter et al. (2000) trained four patients with alcohol-induced amnesia who presented short-term memory deficits, especially for faces and names, to recognize which of two faces matched a previously seen face. The results showed better recognition memory when patients performed the task under the DOP than under non-differential outcomes, a control condition in which the reinforcers are administered according to correct choice but randomly with respect to the correct sample-response pairings (hereafter the NOP condition). Similar DOP benefits have been reported in elderly people that show the typical memory decline associated to aging (López-Crespo et al., 2009), in children and adults with Down syndrome (Esteban et al., 2014) and in patients with Alzheimer’s disease (Plaza et al., 2012). Finally, it has also proved its potential in transient memory problems as those derived from sleep-deprived conditions (Martella et al., 2012).

### The Current Study

The DOP benefit is often accounted for in terms of expectancies functioning as prospective memory representations activated by the to-be-memorized stimuli for which the outcomes will be forthcoming (Savage, 2001). Those expectancies provide an additional source of information so that performance is less affected by longer delays or greater load in working memory. Accordingly, the DOP might be easily adapted to be used in health promotion contexts, where loss of memory about clinical recommendations seems to be on the basis of non-adherence to treatments, mainly in people with memory deficits. Thus, before applying this procedure in real health promotion situations, where patients are required to adhere to medical recommendations, we first conducted a simulated study. We aimed to investigate the potential efficacy of the DOP under conditions that simulate a context where “patients” are required to take different drugs due to multiple morbidities. We assumed that the complexity of the treatment usually taxes people with working memory deficits (e.g., the elderly). To simulate the memory burden that taking several drugs may impose in a multiple morbidity situation, we ran two experiments in which young participants were “prescribed” a treatment that required taking 6 different drugs. To assess the long-term effects of the DOP, participants were also tested 1 h and 1 week after completion of the learning phase. The DOP was expected to help participants to follow the correct treatment recommendations and to ameliorate the detrimental effects of time on memory.

## Experiments 1A and 1B

In the experiments participants were informed that they were participating in a study where they had to learn to associate six different disorders with their respective pills. In Experiment 1A performance in learning the disorder-pill associations and later recognition memory tests of the learned associations, was compared when we employed the DOP and when we employed the non-differential outcomes procedure (NOP). In the NOP, the control condition used here, the outcomes (reinforcers) were the same as those in the DOP, but they were administered in a random way. In Experiment 1B, we replicated the task used in Experiment 1A but required the participants of both DOP and NOP conditions to reach the criterion of 75% correct in the learning phase before the memory testing. Thus, we assessed the benefits of the DOP in long-term retention of the previously learned associations once participants of both groups had reached similar levels of learning. We hypothesized that participants would show better overall performance when trained under the DOP condition than when trained under the NOP condition (higher accuracy in Experiment 1A and less trials to reach the established criterion in Experiment 1B). We also expected to find a higher long-term retention of the learned disorder/pill associations for the DOP group in both experiments.

## Materials and Methods

### Participants

Twenty-one and twenty-seven undergraduate students participated in Experiments 1A and 1B, respectively, in partial fulfillment of a course requirement. Participants ranged in age from 18 to 30 years. Written informed consent was obtained from all participants. The study was approved by the Ethics Committee of the University of Almería, and was conducted in accordance with the approved guidelines and the Declaration of Helsinki. Participants had normal or corrected-to-normal vision and none had previous experience with the learning procedure.

### Stimuli and Materials

The stimuli consisted of the names of six common disorders (high cholesterol, high blood pressure, migraine, hyperglycaemia, hyperthyroidism, and poor circulation) that served as the sample stimuli; the pictures of six different pills that served as the comparison stimuli; and the pictures of six different landscapes that served as the secondary reinforcers. The picture of each secondary reinforcer was displayed along with the phrase “You may win a” followed by the name of a primary reinforcer (desk diary, table game, CD wallet, pack of CDs, pendrive, or book). Primary reinforcers were raffled off at the end of the experiment. Previous studies have demonstrated that raffling of the primary reinforcers is an effective way of assessing the effects of the DOP (e.g., Miller et al., 2002; López-Crespo et al., 2009; Martella et al., 2012; Plaza et al., 2012).

The stimuli were displayed on a white background on a color screen (15-inch VGA monitor) of an IBM-compatible computer. The E-prime program (Psychology Software Tools, 2012) controlled the stimulus presentation as well as data collection.

### Procedure

Participants were tested individually in a quiet room. The experiment consisted of two phases, the learning phase and the memory phase, which lasted for approximately 15 and 5 min, respectively.

**FIGURE 1:**
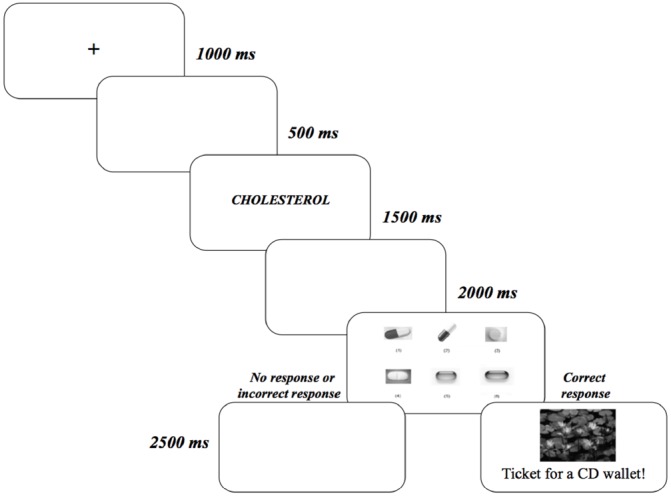
**Stimulus sequence (from left to right) used in Experiments 1A and 1B.** Participants were required to choose the pill (comparison stimulus) that was associated with the disorder (sample stimulus).

In the learning phase of Experiment 1A, participants performed a delayed conditional discrimination task. Participants were instructed that the task required to guess first and then to retain in memory which pill of the six that composed the comparison stimuli was associated with the specific disorder that was previously presented as the sample stimulus. The trial sequence (see Figure 1) began with a central fixation point (an asterisk) for 1000 ms. After an interval of 500 ms, the name of a disorder was displayed for 1500 ms. After an additional interval of 2000 ms, the pictures of the six pills numbered 1–6, were presented during 10 s or until the participant responded. Participants responded by pressing the key that corresponded to the pill number they associated with that particular disorder. The picture of a landscape (the outcome) and the phrase reminding the associated prize followed the correct responses for 2.5 s. Incorrect responses were followed by a blank screen that lasted the same time as the outcome presentation for correct responses. The trial was also scored as incorrect if the participant did not emit any response in 10 s.

Experiment 1A consisted of 96 trials grouped in two blocks of 48 trials each. Participants performed the two blocks of trials with a rest period in between at the participant’s discretion. Once the learning session had been completed, participants were scheduled for the two memory tests, one taking place 1 h later and the other 1 week later. None of the participants was informed in advance that the memory tests would be administered after the learning phase.

Experiment 1B was identical to Experiment 1A with the exception that (i) it consisted of 108 trials grouped in nine blocks of 12 trials each. (ii) The learning phase finished once the participant reached the criterion of 75% correct or above in one block of trials. Participants were also informed that once they achieved the established criterion, or after 108 trials, whichever occurred first, the task would end. (iii) A rest period was provided after every 36 trials so that it could be one or two rest period depending on the participant’s performance.

Participants from both experiments were randomly assigned to one of the two experimental training conditions, the differential outcomes condition (DOP; 10 and 13 in Experiments 1A and 1B, respectively) and the non-differential outcomes or control condition (NOP; 11 and 14 in Experiments 1A and 1B, respectively). Participants in the DOP condition received specific outcomes following correct responses. For instance, for some participants the cholesterol/blue pill association was reinforced with the picture of a sunset and the phrase “You may win a desk diary!”; the migraine/red pill association with the picture of a pine forest in winter and the phrase “You may win a CD wallet!”; the hyperthyroidism/white and red pill association with the picture of some water lilies and the phrase “You may win a table game!,” and so on. Correct responses in the NOP condition were also reinforced but the outcomes were administered in a random way. The disorder/pill associations were randomized across participants.

The memory phase was identical in both experiments. All participants performed the recognition memory tests 1 h and 1 week after the learning phase. Each memory test consisted of six trials, one with each trained disorder-pill association, with the same stimulus sequence that was displayed in the learning phase (see Figure 1). However, in the memory tests we did not administer any outcome following the participant’s response. A trial was scored as correct if the participant chose the pill that was associated with the specific disorder during the learning phase within a time limit of 10 s.

### Statistical Analysis

In Experiment 1A we assessed accuracy differences between the DOP condition and the NOP condition through an inspection of the learning curves. We first grouped the 96 trials in six blocks of 16 trials each. Correct responses were then submitted to a mixed ANOVA with Outcomes (DOP and NOP) as the between-participant factor and Blocks of trials (1, 2, 3, 4, 5, and 6) as the within-participant factor.

In Experiment 1B, the number of trials required by each participant to reach the established criterion was entered into a one-way ANOVA, with Outcomes (DOP and NOP) as the between-participant factor. Data from one participant assigned to the NOP condition were excluded from the statistical analysis because of a failure to reach the criterion.

Finally, percentages of correct responses from the two recognition memory tests were submitted to a mixed ANOVA, with Outcomes (DOP and NOP) as the between-participant factor and Test time (1-h and 1-week) as the within-participant factor. The statistical significance level was set at *p* ≤ 0.05.

## Results

Latency data did not show any significant effect in any of the experiments, and therefore only accuracy data are reported.

### Experiment 1A

**FIGURE 2:**
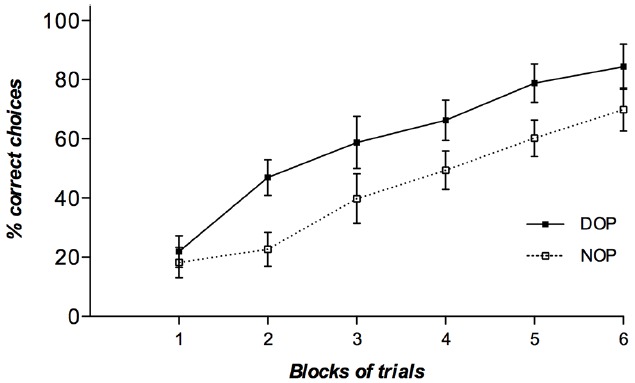
**Mean percentages of correct choice responses obtained by participants in the learning phase of Experiment 1A as a function of blocks of trials (six blocks of 16 trials each) and outcomes (DOP and NOP).** Error bars show the standard error of the mean.

Results from the accuracy data analysis showed significant main effects of both Outcomes [*F*(1,19) = 5.14, *p* = 0.035, ${\text{η}}_{\text{p}}^{\text{2}}$ = 0.21] and Block of trials [*F*(5,95) = 37.17, *p* < 0.001, ${\text{η}}_{\text{p}}^{\text{2}}$ = 0.66]. Participants in the DOP condition showed higher accuracy (59% correct) than participants in the NOP condition (43% correct). Accuracy linearly increased with blocks of trials (20, 62, 64, 75, 75, and 79% correct in blocks 1, 2, 3, 4, 5, and 6, respectively). Although the two-way interaction did not reach significance [*F*(5,95) < 1] it is apparent from Figure 2 that differences in the learning curves between the DOP and the NOP conditions emerged from block 2 and on. In fact, the DOP and NOP conditions did not differ significantly in the first block of trials [*t*(19) = 0.53, *p* = 0.62].

### Experiment 1B

The analysis of the number of trials needed to reach the established criterion (a minimum of 75% correct in one block of trials) revealed a significant main effect of Outcomes [*F*(1,24) = 5.24, *p* = 0.03, ${\text{η}}_{\text{p}}^{\text{2}}$ = 0.18]. Participants in the DOP condition required fewer trials (42 trials) than participants in the NOP condition (58 trials) to reach the criterion. Importantly, there were no significant differences in the overall accuracy obtained by participants from both conditions in the learning task (55 vs. 54% correct in the DOP and NOP conditions, respectively; *p* > 0.05).

### Experiments 1A and 1B: Recognition Memory

Table 1 shows the percentage of correct responses in the memory tests. Only 11 participants from Experiment 1A (5 from the DOP condition and 6 from the NOP condition) and 19 participants from Experiment 1B (9 from the DOP condition and 10 from the NOP condition) completed both tests.

**TABLE 1:** **Mean percentages of correct responses and standard error of the mean (SEM; in parentheses) in the memory recognition tests as a function of Outcomes (DOP: differential outcomes procedure, NOP: non-differential outcomes procedure) and Time of testing (1 h, 1 week). Experiments 1A and 1B**.

	**1 hour**		**1 week**	
	**DOP**	**NOP**	**DOP**	**NOP**
Experiment 1A	87% (16.64)	50% (10.51)	77% (15.19)	36% (9.6)
Experiment 1B	70% (12.6)	62% (11.95)	75% (8.85)	35% (8.39)

In Experiment 1A, the results revealed a significant main effect of Outcomes [*F*(1,9) = 5.22, *p* = 0.048, ${\text{η}}_{\text{p}}^{\text{2}}$ = 0.37]. That is, participants showed better retention of the learned associations when trained with differential outcomes (82% correct) than with non-differential outcomes (43% correct). Although recognition memory decreased from 1-h to 1-week in both outcomes conditions (see Table 1), such reduction did not reach statistical significance (*p* > 0.05).

In Experiment 1B, the results showed that the only statistically significant effect was the Outcomes × Test time interaction [*F*(1,17) = 5.04, *p* = 0.038, ${\text{η}}_{\text{p}}^{\text{2}}$ = 0.23]. Although recognition memory was lower with the NOP condition in both delays, the difference between the DOP condition and the NOP condition was significant only in the 1-week test [*F*(1,20) = 4.81, *p* = 0.040, ${\text{η}}_{\text{p}}^{\text{2}}$ = 0.19].

Importantly, when we combined the results from both experiments the long delay affected recognition memory greatly and significantly in the NOP condition (20.5 points) [*t*(15) = 2.6, *p* = 0.02], and barely and non-significantly in the DOP condition (2.5 points) [*t*(13) = 1.09, *p* = 0.92].

## Discussion

Success in the treatment and control of many diseases depends on patient adherence to the medical recommendations, including implementing any specific medications (Jimmy and Jose, 2011). Currently non-adherence to medication is a serious problem, as it has been noted by the World Health Organization (2003) that between 30 and 50% of medicines prescribed for chronic illnesses are not taken as intended. Consequences of non-adherence include substantial worsening of disease, severe relapses, increased comorbid diseases, death, and increased health care costs (Chisholm-Burns and Spivey, 2003; Peterson et al., 2003). Although non-adherence results from many causes, one of the major reasons for non-adherence to medical recommendations is that patients forget to take their medications as they were prescribed (Jimmy and Jose, 2011). This might be especially true for a population usually associated with a low adherence to medications (Kripalani et al., 2007), namely, elderly people who have multiple morbidities that must be treated simultaneously with several medications. Their impaired short-term memory and their decrease working memory capacity (Cavanaugh and Blanchard-Fields, 2006) could result in older adults forgetting the medication associated with a specific disease or the scheduled time for administration, as well as the correct dosage of such medication. Thus, any procedure that helps patients (especially those with memory impairments) to ameliorate their memory loss concerning medical treatment prescriptions by increasing, for example, their ability to choose the correct medicine, might have a great impact in their adherence to medical recommendations and hence in their health.

In the present study we have illustrated the potential benefits of the DOP, a procedure that has proved its usefulness to improve both learning and memory of symbolic relationships in different populations. Concretely, we aimed to explore whether healthy adults may learn symbolic associations between stimuli faster and with better long-term retention when trained with the DOP in a health promotion context. To simulate a situation in which “patients” with memory deficits (e.g., elderly people) are told to take different drugs due to multiple morbidities, our participants had to associate six common diseases with their correspondent treatments (six different pills), a condition that clearly taxes working memory. The results observed in the two experiments showed better performance (higher accuracy in Experiment 1A and fewer trials to reach the criterion in Experiment 1B) when differential outcomes were arranged (the DOP condition) compared with when the same outcomes were randomly administered (the NOP condition). Importantly, the present findings demonstrate that the use of such a simple technique facilitates not only the learning of symbolic stimulus–stimulus relationships but also the long-term memory retention of the learned associations. The advantage of the DOP compared with the NOP in the shorter delay test was more evident in Experiment 1A than in Experiment 1B, maybe due to participants in the former experiment having more trials to consolidate the learned associations than participants in the latter experiment. However, when the learning level was equated between the two outcomes conditions (Experiment 1B) the DOP advantage was still observed. Therefore, the results observed in the memory tests cannot be attributed to participants from the DOP condition producing more correct responses on average, and therefore reaching better learning consolidation compared with participants in the NOP condition. Importantly, when we combined the results from both experiments it was apparent that recognition memory was only affected by the long delay in the NOP condition but not in the DOP condition. This finding can be due to the DOP condition improving both the learning and long-term retention of what had been previously learned.

It is also worth noting that in the current study participants had to perform a complex learning task that required to associate six different stimulus-outcome pairings, more than the 2–4 different stimulus-outcome pairing typically reported in the DOP’s literature (e.g., Maki et al., 1995; Estévez et al., 2001, 2003, 2007; Martínez et al., 2012). Thus, the fact that we have observed DOP beneficial effects on both learning and memory using such high number of pairings is further evidence of its potential as a technique to improve both processes in clinical and non-clinical settings.

The superiority of the DOP has been explained by the expectancy theory (Trapold and Overmier, 1972). When participants perform a conditional discrimination task they learn something specific about the properties of the outcomes. Trained with the DOP participants develop unique expectancies (or prospective memories) that become discriminative stimuli that serve as additional guides for choice behavior. In the non-differential condition expectations are also generated, but they are common to all sample stimuli, and then participants can rely only on retrospective memory of the sample stimulus to perform correctly the task, a process affected by working memory load and delays.

In summary, the present findings demonstrate that the use of specific outcomes associated with each symbolic relation in a discriminative task, improves not only the acquisition of learning but also its long-term retention in healthy adults. It is worth noting that in this study we simulated daily activity of many elderly people that have to associate determined pills to the particular disorder they treat. The ecological nature of this symbolic discrimination task, allows us to highlight the therapeutic potential of the DOP as a complementary strategy for people with learning and memory deficits (e.g., those with neurodegenerative disorders associated with aging such as patients with Alzheimer’s or Parkinson’s disease), which seriously affect their daily life. Techniques based on the DOP are easy to set up, and may facilitate the learning and retention of relevant information that will enhance people’s quality of life. The results of our simulated study suggest that the DOP can be used as a useful complement to other intervention programs targeted to increase adherence to treatment.

## Author Contributions

MM was responsible for data collection and participated in statistical analysis, and manuscript preparation. VP contributed to the design of the study and manuscript preparation. LF and AF contributed to data interpretation and assisted with writing the manuscript. AF was also responsible for the design of the study.

### Conflict of Interest Statement

The authors declare that the research was conducted in the absence of any commercial or financial relationships that could be construed as a potential conflict of interest.
